# Analysis of an Intervention for Emergency Medical Services Personnel to Reduce Epinephrine Dosing Errors in Infants

**DOI:** 10.1001/jamanetworkopen.2022.7645

**Published:** 2022-04-15

**Authors:** Matt Hansen, Grace Walker-Stevenson, Carl Eriksson, Garth Meckler, Tabria Harrod, Nathan Bahr, Jeanne-Marie Guise

**Affiliations:** 1 Departments of Emergency Medicine and Pediatrics, Oregon Health & Science University, Portland; 2 Department of Obstetrics and Gynecology, Oregon Health & Science University, Portland; 3 Department of Pediatrics, Oregon Health & Science University, Portland; 4 Division of Pediatric Emergency Medicine, British Columbia Children’s Hospital, Vancouver, British Columbia, Canada

## Abstract

This quality improvement study investigates whether a simulation-driven emergency medical services protocol of using a 1-mL syringe to administer small epinephrine doses could reduce dosing errors in infants.

## Introduction

Several studies have documented epinephrine dosing errors in out-of-hospital cardiac arrest resuscitation of pediatric patients.^1,2^ Currently, adults receive a 1-mg dose of epinephrine during a cardiac arrest.^3^ The pediatric dose is 0.01 mg/kg (0.1 mL/kg), which can be calculated using the patient’s actual weight or estimated based on age or length. The smallest children are at the greatest risk of epinephrine dosing errors, owing to the potential for decimal translation errors (resulting in 10-fold errors) and the inaccuracy in delivering smaller doses using commonly supplied (1 mg/10 mL) prefilled epinephrine syringes.^4^ Infants also account for approximately 40% of pediatric out-of-hospital cardiac arrests.^5^ Large overdoses of epinephrine are associated with poorer survival.^4^ Although incorrect epinephrine dosing is known to be a problem, there is currently little evidence regarding how to mitigate it. This study investigated whether a simulation-driven emergency medical services (EMS) protocol change using a 1-mL syringe to administer small epinephrine doses reduces dosing errors in infants.

## Methods

In this quality improvement study, we conducted simulations in 2 different time periods and we collected data in real time during the simulation scenarios. All aspects of the study were approved by the Oregon Health & Science University Institutional Review Board. Informed consent was obtained from all participants. The study followed the SQUIRE reporting guidelines.

All simulations were conducted in situ using the same infant simulator and the same EMS agencies in a large regional EMS system in which advanced life support–capable public fire and private transfer units respond to all calls for service.

In 2016, we conducted a neonatal resuscitation simulation that included indications for intravenous administration of 1 mg/10 mL of epinephrine. Medication dosing was measured by clinical experts with a standardized tool using both real-time observation and video review. Simulations included real-time didactic debriefings. We communicated lessons learned to the EMS medical directors after the study. Specifically, to improve the accuracy of dosing and reduce the magnitude of potential overdoses, we recommended that agencies draw up epinephrine doses of 1 mL or less in a 1-mL syringe (eFigure in the Supplement) rather than use a (1 mg/10 mL) prefilled epinephrine syringe.

In 2020, we conducted additional simulation sessions including a 4-month-old infant in nonshockable cardiac arrest requiring epinephrine. EMS pediatric training hours were unchanged from normal during the study period.

The correct epinephrine dosage was defined as ±20% of the ideal dose calculated using the simulator length to estimate infant weight.^6^ The dose recommended by the local length-based guide was within this range. We performed a 2-tailed Fisher exact test to compare the 2016 and 2020 data; this test was selected because of our relatively small sample sizes. All statistical analyses were performed using Stata version 15.

## Results

A total of 432 EMS personnel participated in this study. In 2016, we conducted 47 neonatal cardiac arrest simulations (209 EMS personnel), which included approximately 15% of the area EMS workforce (1400 personnel). Two simulations were excluded because of audio/video problems and lack of participant consent. In 2020, we conducted 39 infant cardiac arrest simulations (223 EMS personnel). As shown in Table 1, the percentage of patients who received epinephrine increased significantly from 2016 (19 of 45 [42%]) to 2020 (36 of 39 [92%]) (*P* < .001). Among those who received epinephrine, the percentage who received an appropriate dose also increased significantly from 2016 (12 of 19 [63%]) to 2020 (35 of 36 [97%]) (*P* = .01; Table 2). Time to epinephrine delivery decreased from a mean (SD) of 395 (30) seconds in the 2016 simulations to 322 (18) seconds in the 2020 simulations using the 1-mL syringe (*P* < .001).

**Table 1.** Epinephrine Administration by Simulation Year^a^

Epinephrine dose	No. of infants who received epinephrine	
	2016 (n = 45)	2020 (n = 39)
None	26	3
Any	19	36

^a^ *P* < .001 by Fisher exact test for comparison of the total number of infants who received epinephrine in 2016 vs 2020.

**Table 2.** Epinephrine Dose Category by Simulation Year^a^

Epinephrine dose^b^	No. of infants who received epinephrine	
	2016 (n = 17)^c^	2020 (n = 36)
Correct	12	35
Underdose	2	0
Overdose		
<10×	2	0
10×	1	1

^a^ *P* = .01 by Fisher exact test for comparison of the total number of infants who received epinephrine in 2016 vs 2020.

^b^ Correct doses were considered to be ±20% of the recommended dose.

^c^ In 2016, we had 2 doses with unknown amounts; therefore, they were excluded from these calculations.

## Discussion

In this simulation-based quality improvement study, a simple EMS intervention using a 1-mL syringe for small epinephrine doses was associated with an improved rate of administering the correct epinephrine dose without slowing delivery speed. The limitations of this study include its observational design and single urban EMS system setting. These findings suggest that a similar intervention could reduce epinephrine dosing errors among infants.

## Notes

Supplement. **eFigure.** Method Using a 1-mL Syringe to Draw Epinephrine From a 10-mL Prefilled Syringe via a 3-Way Stopcock
