# Supplementary material for: Analysis of an Intervention for Emergency Medical Services Personnel to Reduce Epinephrine Dosing Errors in Infants
Source: JAMA Netw Open. 2022 Apr 15;5(4):e227645. doi: 10.1001/jamanetworkopen.2022.7645 (PMC9012966; doi:10.1001/jamanetworkopen.2022.7645)
Supplement: Supplement. — eFigure. Method Using a 1-mL Syringe to Draw Epinephrine From a 10-mL Prefilled Syringe via a 3-Way Stopcock [file jamanetwopen-e227645-s001.pdf]

Supplementary Online Content

Hansen M, Walker-Stevenson G, Eriksson C, et al. Analysis of an intervention for emergency medical services personnel to reduce epinephrine dosing errors in infants. *JAMA Netw Open*. 2022;5(4):e227645. doi:10.1001/jamanetworkopen.2022.7645

eFigure. Method Using a 1-mL Syringe to Draw Epinephrine From a 10-mL Prefilled Syringe via a 3-Way Stopcock

This supplementary material has been provided by the authors to give readers additional information about their work.

© 2022 Hansen M et al. *JAMA Network Open*.

eFigure. Method Using a 1-mL Syringe to Draw Epinephrine From a 10-mL Prefilled Syringe via a 3-Way Stopcock

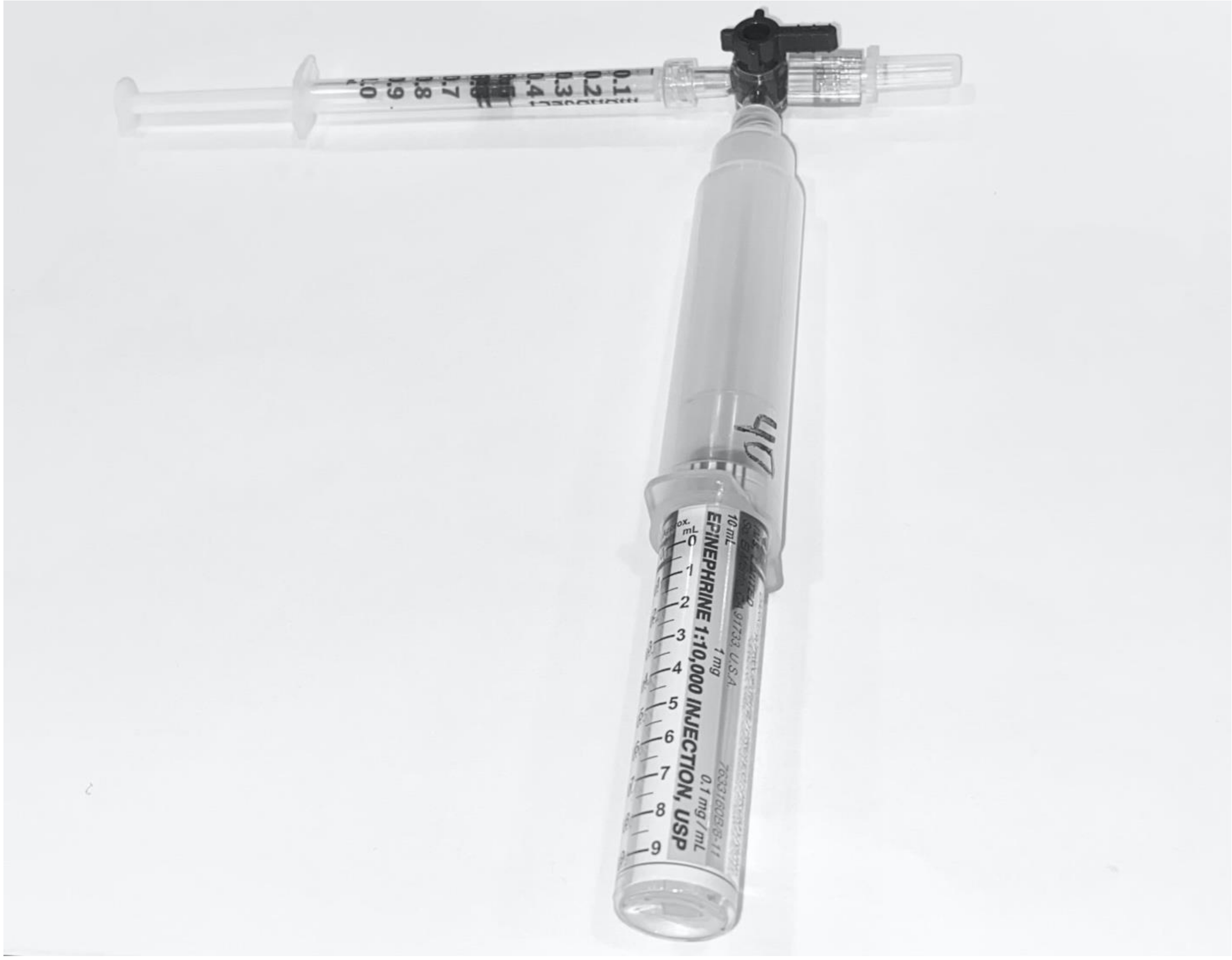

© 2022 Hansen M et al. *JAMA Network Open*.
